# Co-prevalence of extracranial carotid aneurysms differs between European intracranial aneurysm cohorts

**DOI:** 10.1371/journal.pone.0228041

**Published:** 2020-01-23

**Authors:** Constance J. H. C. M. van Laarhoven, Vanessa E. C. Pourier, Antti E. Lindgren, Mervyn D. I. Vergouwen, Juha E. Jääskeläinen, Gabriël J. E. Rinkel, Dominique P. V. de Kleijn, Gert J. de Borst

**Affiliations:** 1 Department of Vascular Surgery, University Medical Center Utrecht, Utrecht University, Utrecht, the Netherlands; 2 Department of Neurosurgery, NeuroCenter, Kuopio University Hospital, Kuopio, Finland; 3 Brain Center Rudolf Magnus, Department of Neurology and Neurosurgery, University Medical Center Utrecht, Utrecht University, Utrecht, the Netherlands; Universitatsklinikum Freiburg, GERMANY

## Abstract

**Background and purpose:**

Previously, we showed that co-prevalence of extracranial carotid artery aneurysms (ECAAs) in patients with intracranial aneurysms (IAs) was 2% in a Dutch cohort. In order to obtain more precise estimates and discover potential predictors of ECAA co-prevalence in the European population, we retrospectively compared differences and similarities of our Dutch cohort with a Finnish cohort using protocolled imaging of the cerebrovascular tree.

**Methods:**

IA patients within the prospective database of the Kuopio University Hospital were eligible for this study (n = 1,118). Image analysis and hospital chart review were conducted.

**Results:**

In total, 458 patients with complete carotid imaging conform protocol were analyzed. Twenty-four ECAAs in 21 patients were identified (4.6%, 95% CI 2.9–6.9), a higher co-prevalence than in the Dutch cohort (1.9%; 95% CI 1.0–3.3), prevalence odds ratio (POR) 2.45 (95% CI 1.19–5.03). In the Finnish cohort, 25% of all ECAAs were located around the carotid bifurcation, others in the internal carotid artery distally from the bifurcation. Independent predictors for ECAA co-prevalence were origin of country (POR 2.41, 95% CI 1.15–5.06) and male gender (POR 2.25, 95% CI 1.09–4.64).

**Conclusion:**

The co-prevalence of ECAA in IA patients was twice as high in the Finnish compared to the Dutch IA cohort, with origin of country and male gender as independent predictors. Twenty-five percent of ECAAs would be missed, if the carotid bifurcation was not imaged. Therefore, we propose to always include imaging of the carotid bifurcation as the gold standard technique to identify ECAA in IA patients.

## Introduction

The extracranial carotid artery aneurysm (ECAA) is a rare vascular entity that accounts for less than 1% of all peripheral artery aneurysms. [1–5] The majority of patients is asymptomatic, and the carotid aneurysm is often found by coincidence. If symptomatic, most observed symptoms are cervical complaints like pain, mass or thrill, and nerve palsies due to local compression by the dilated carotid artery. A smaller proportion of patients presents with cerebral ischemia, *i*.*e*. transient ischemic attack or stroke. [4–7] Due to the rarity of the disease, comprehensive literature and clinical guidelines are lacking. [8]

Since peripheral aneurysms share several risk and genetic factors, co-prevalence of arterial aneurysms within different vascular beds is commonly seen. [9–15] Although the evidence level for these findings are low and benefits of screening under debate, radiological screening for other (abdominal aortic) aneurysms may be indicated when patients are presented with a peripheral aneurysm. [16] In this light, our research group previously investigated co-prevalence of intracranial aneurysms (IAs) and ECAA in a selected Dutch IA cohort. [1] Due to the non-standardized and heterogeneous radiologic carotid imaging in IA patients, the reported co-prevalence of ~two percent of ECAA in IA patients, might be an underestimation.

In the present study, we studied another European IA cohort operating with a standard imaging protocol since 2007 including the aortic arch up to top of the brain by at least CTA or MRA. The primary aim was to compare the co-prevalence of ECAA in IA patients in the Finnish and Dutch cohort. Secondly, we combined the two cohorts to adjust for confounding and possibly identify independent predictors for ECAA presence.

## Materials and methods

### Patient population

Following approval of the Institutional Research Ethics Board of the Kuopio University Hospital (KUH) for this retrospective study, we analyzed data from a prospective database (http://www.kuopioneurosurgery.fi/database) with patients admitted for IA to the KUH in Kuopio, Finland, from January 2010 to December 2016. All participants gave informed consent for this prospective database. All research was conducted according to the principles of the Declaration of Helsinki and its later amendments. The KUH is a tertiary referral center and the sole provider of neurosurgical services in its geographical catchment area (approximately 850,000 inhabitants) allowing prospective collection of a population-based IA cohort. Patients aged 18-years or older and with a radiologically confirmed IA were included. Exclusion criteria were: no extracranial carotid arterial imaging available, IAs caused by a trauma, arteriovenous malformation, cavernous malformation, dural fistula or cerebral venous sinus thrombosis. Crude data from a similar Dutch study performed in IA patients from the University Medical Center Utrecht (UMCU) were obtained (S1 File). [1]

### Data collection

All available cerebrovascular imaging was reviewed and categorized as complete or incomplete carotid imaging. Complete imaging of the cerebrovascular tree was defined as imaging from aortic arch up to top of the brain, depicting the common, internal, external and intracranial carotid arteries (*i*.*e*. CTA stroke or carotids). Imaging of the cerebrovascular tree was considered incomplete if imaging included only the distal (*i*.*e*. CTA Willis) part of the external and internal carotid artery (ICA), mostly from the second cervical vertebra up to the vertex. Patients undergoing CTA evaluation were scanned with 64-slice CT scanners, MRA imaging was performed on 1.5 or 3.0 Tesla scanners. Imaging was performed for a variety of reasons, including follow up of invasive or conservative treatment of the IA, stroke, dementia, or e.g. headache. All of the scans were reviewed on Sectra AB/PACS Software (Linköping, Sweden).

The diagnosis of IA was determined according to KUH IA database records. The diagnosis of ECAA was determined by reviewing the available radiological images of each patient and the original radiology reports. Fusiform or spindle-shaped ECAA was defined as ≥150% dilation of the arterial diameter, compared with the non-affected contralateral carotid artery diameter. In case of bilateral dilatation, the diameter of the non-affected part of the ipsilateral carotid artery was used as comparison. For saccular shaped ECAA, all sizes were accepted [1,7]. Location of ECAA was divided in proximal ECAA, around the carotid bifurcation, and distal ECAA. Proximal was defined as any location within the common carotid artery (Attigah type 5), and distal as any location in the internal carotid artery (Attigah type 1 and 2). If the ECAA location affected the carotid bifurcation, this was scored as around the carotid bifurcation (Attigah type 3 and 4). [17] ECAA-related symptoms and presumed etiology of the ECAA were retrieved from the hospital records. The side (left or right), arterial site of the carotid artery, shape and diameter of ECAA were retrieved from available radiological reports. In case the size was not reported, two authors (CL, AL) independently measured the maximum aneurysm diameter on available examinations. Any disagreement in scoring was discussed with an experienced third and fourth independent observer (VP, GB) until final agreement was reached.

A hospital chart review was conducted for each patient to identify comorbidities and risk factors. Hypertension was defined as blood pressure of ≥140/90 mmHg and/or use of antihypertensive medication, diabetes as any use of antidiabetic medication, hyperlipidemia as any use of blood lipid lowering drugs, cardiac disease was defined as any cardiac event (e.g. myocardial infarction, arrhythmia, or cardiac intervention like percutaneous coronary intervention), polycystic kidney disease was scored only if radiologically confirmed, and rheumatoid arthritis if stated within medical records. Smoking was defined as current smoking at time of ECAA diagnosis. Family history of IA was defined as >1 first degree relative with IA.

### Outcome and statistical analysis

The primary outcome was defined as the co-prevalence of IA and ECAA in the Finnish cohort, as confirmed in the KUH radiology report. We compared the co-prevalence of the Finnish and Dutch cohort. Second, we combined the two cohorts with the aim to identify independent predictors for ECAA presence, analyzing only patients with complete carotid imaging, to ensure that the primary outcome is reliably measured.

Differences were tested with Student two-tailed *t* test and χ^2^ test when appropriate. For the primary analysis, the total number of ECAA patients was divided by the total amount of patients within the sample, and multiplied with 100, and 95% CI [18] were calculated. The secondary outcome was assessed by multiple logistic regression. Potential confounders were selected based on a univariate analysis (*p<*0.1) and literature. [1–6] Prevalence odds ratios (POR) and corresponding 95% CI were calculated, and significance was set at *p*<0.05. [19] All statistical analyses were performed using SPSS software version 25.0 for Windows (SPSS Inc, Chicago, Illinois, USA).

### Results and discussion

**Fig 1:**
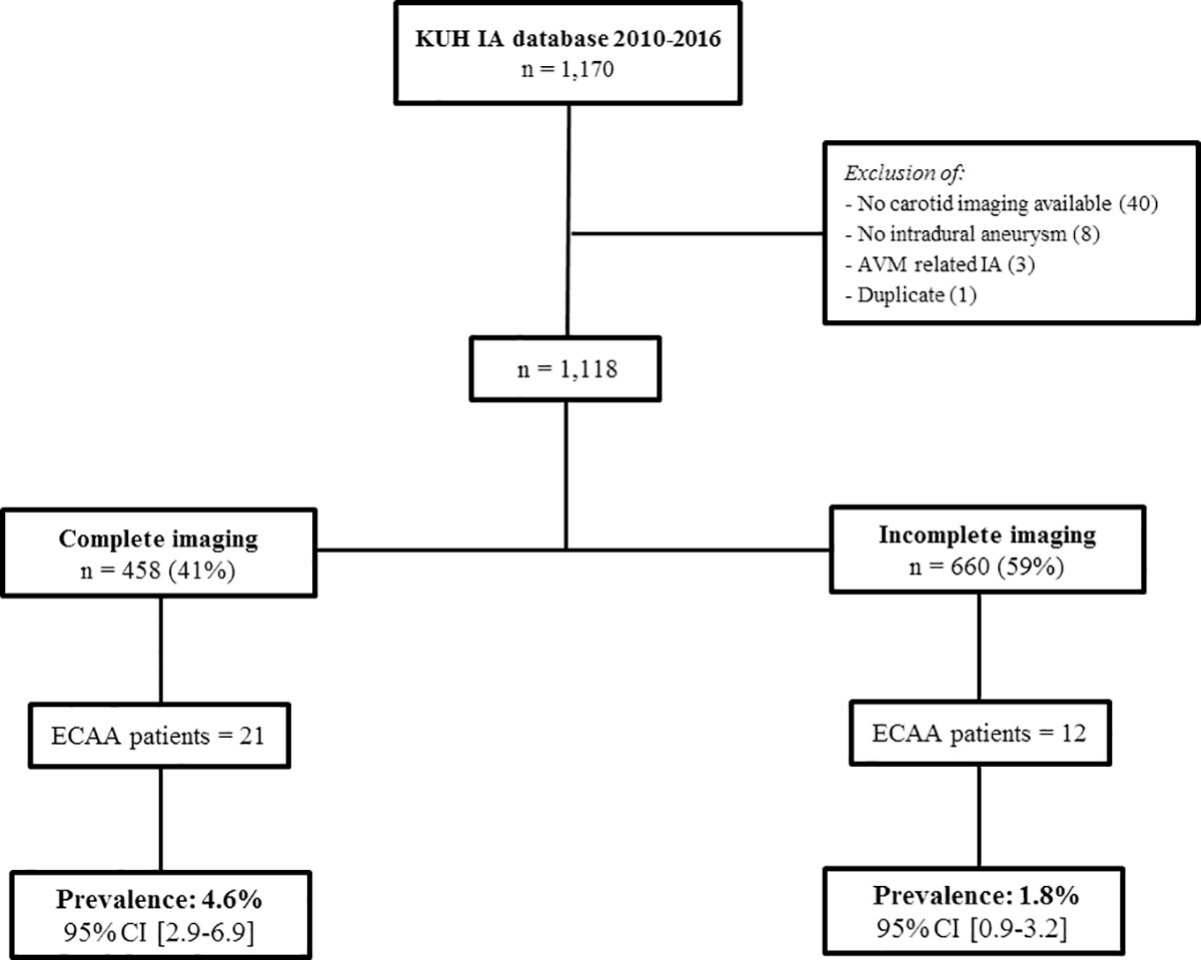
Flowchart of Finnish cohort. KUH = Kuopio University Hospital, IA = intracranial aneurysm, n = number of patients, AVM = arteriovenous malformation, ECAA = extracranial carotid artery aneurysm.

In total, 1,170 IA patients were available from the KUH database. After exclusion of patients without available carotid imaging (n = 40), absence of intradural aneurysms (n = 8), AVM related IA (n = 3) and one duplicate, 1,118 patients remained for analysis (see Fig 1). In 458 (41%) patients, the cerebrovascular tree was completely imaged (CTA 91%, MRA 9%) conform protocol, while 660 (59%) patients had incomplete carotid imaging.

### Comparison of Finnish and Dutch cohort

**Fig 2:**
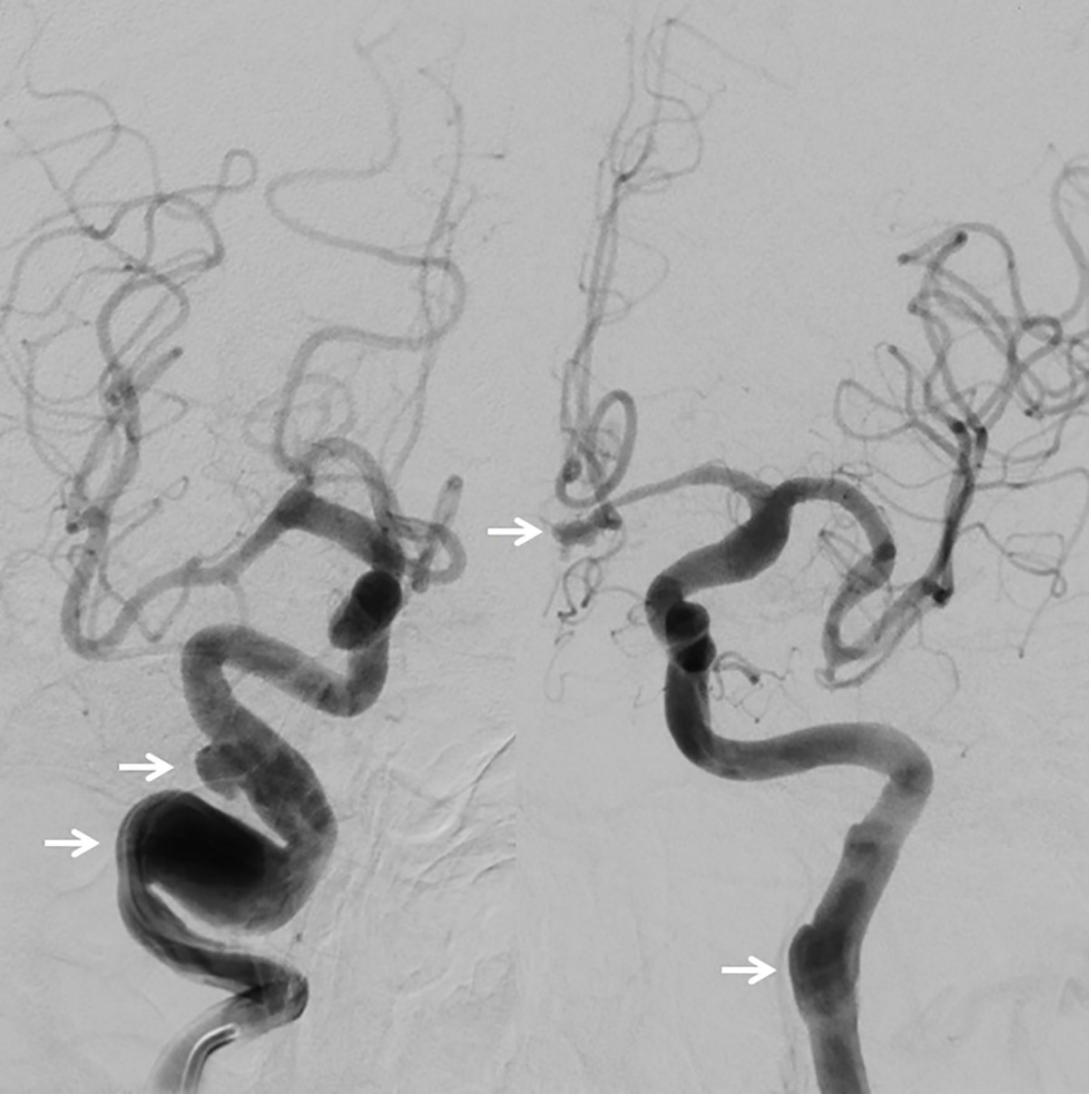
Overview DSA brain of an asymptomatic patient in posterior-anterior configuration. *Left*: a fusiform ECAA in the right ICA and saccular shaped ECAA in the distal part of the RICA. *Right*: a fusiform dilated ECAA in the left ICA and a saccular shaped IA of the anterior communicating artery (all indicated by white arrows). *Abbreviations*: DSA = digital subtraction angiography, ECAA = extracranial carotid artery aneurysm, ICA = internal carotid artery, IA = intracranial aneurysm.

**Fig 3:**
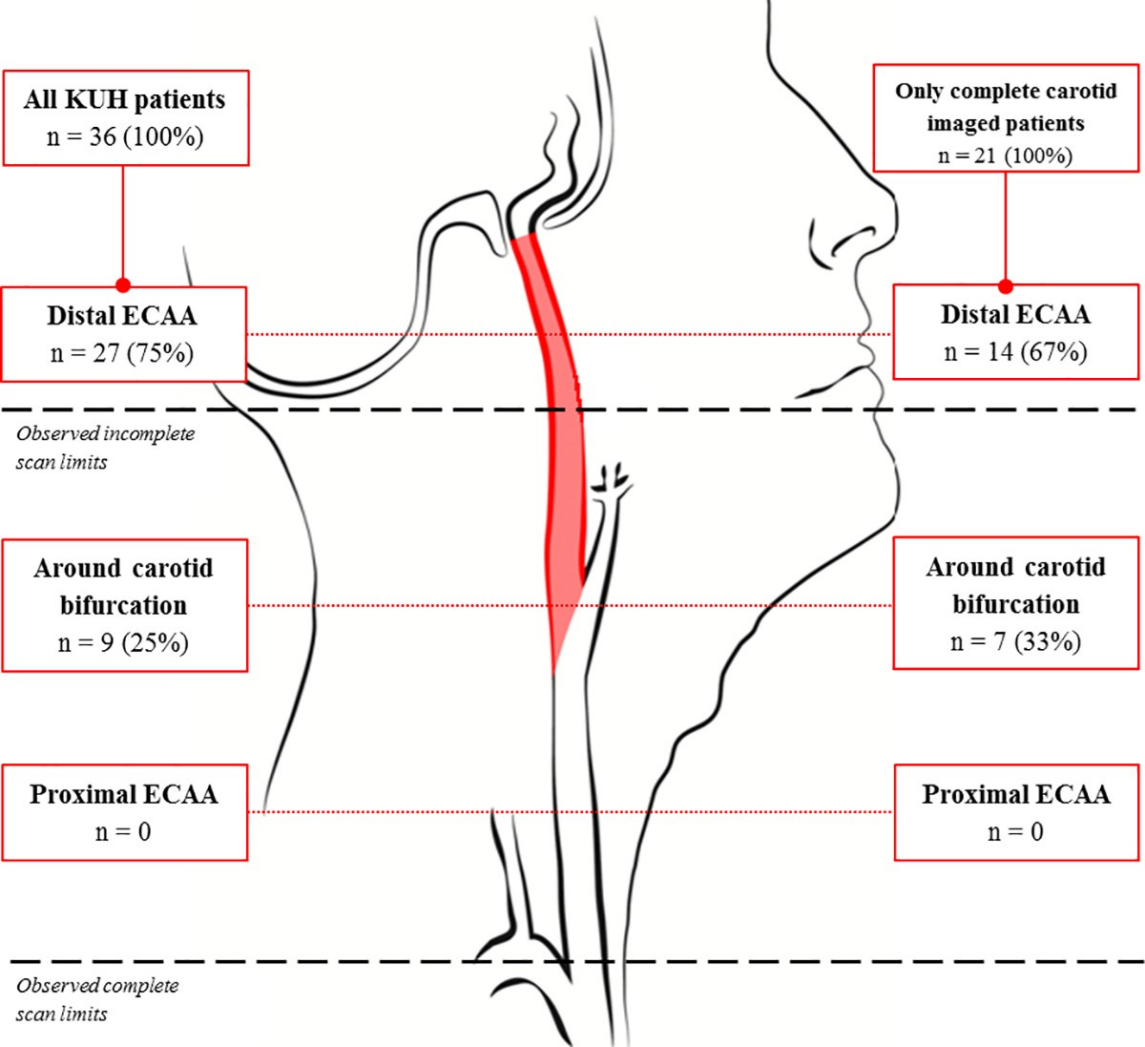
Schematic overview of location of 36 ECAAs in the extracranial carotid artery of the Finnish cohort. *Abbreviations*: KUH = Kuopio University Hospital, ECAA = extracranial carotid artery aneurysm.

In the total Finnish cohort, we identified 36 ECAAs in 33 Finnish IA patients (Fig 1). In the complete carotid imaging, 24 ECAAs in 21 patients were identified (Fig 2), which corresponds with a Finnish prevalence of 4.6% (21/458), 95% CI 2.9–6.9. In the incomplete carotid imaging patients, only 12 patients with an ECAA were identified, resulting in a lower Finnish prevalence of 1.8% (12/660), 95% CI 0.9–3.2. Detailed ECAA characteristics are summarized in S1 Table. In the Finnish cohort, all ECAAs were located in the ICA, divided in twenty-seven (75%) distal ECAA, and nine (25%) around the carotid bifurcation (Fig 3).

The comparison of the co-prevalence of only the complete carotid imaging patients in the Finnish (4.6%) and Dutch cohort (1.9%), showed an unadjusted significant difference, *p =* 0.019, POR 2.45 95% CI 1.19–5.03. For further analysis, we only include patients with complete carotid imaging.

### Identification of clinical predictors

The baseline characteristics of both the Dutch and Finnish cohort are summarized in Table 1. The cohorts differed in sex, age, hypertension, diabetes and statin use. In Table 2, the univariate screen for factors associated with ECAA prevalence is shown. Besides sex (*p* = 0.021) and origin of cohort (*p* = 0.019), we included age, hypertension, diabetes and any statin use (Table 1) in the multiple logistic regression analysis. Both origin of country (POR 2.41, 95% CI 1.15–5.06) and male gender (POR 2.25, 95% CI 1.09–4.64) remained as independent factors for ECAA prevalence (Table 3).

**Table 1:** Description of the study population with complete carotid imaging of the Finnish and Dutch cohort.

	Finnish n = 458		Dutch^a^ n = 624		*p*
	n	(%)	n	(%)	
Male gender	208	(45)	222	(36)	0.001*
Age at admission (mean, sd) in years	61	11.9	59	12.4	0.003*
Hypertension	214	(49)	241	(39)	0.004*
Cardiac disease	90	(20)	105	(17)	0.318
ADPKD	3	(1)	8	(1)	0.461
Rheuma	12	(3)	19	(3)	0.784
Diabetes	57	(13)	28	(5)	0.000*
Family history IA	27	(6)	26	(6)	0.935
Smoking, current	141	(41)	210	(37)	0.175
Statin use	216	(47)	174	(29)	0.000*
Presentation SAH	175	(38)	264	(42)	0.196

n = number, sd = standard deviation, ADPKD = autosomal dominant polycystic kidney disease, IA = intracranial aneurysm, SAH = subarachnoid hemorrhage.

* indicates *p* < 0.05.

^a^ Crude data from *Pourier et al*. [1]

**Table 2:** Univariate screen for factors associated with ECAA prevalence.

		ECAA n = 33^a^		No ECAA n = 1049		*p*
		n	(%)	n	(%)	
Age at admission (mean, sd) in years		59.7	12.2	56.9	14.3	0.198
Gender						0.021^*^
	Male	20	(61)	410	(39)	
	Female	13	(39)	639	(61)	
Origin of cohort						0.019^*^
	Finland	21	(64)	437	(42)	
	The Netherlands	12	(37)	612	(58)	
Hypertension		16	(48)	439	(42)	0.665
Cardiac disease		7	(21)	188	(18)	0.828
ADPKD		0	-	11	(1)	-
Rheuma		2	(6.1)	29	(2.8)	0.565
Diabetes		2	(6.1)	83	(7.9)	0.937
Family history IA		1	(3.0)	52	(5.0)	0.862
Smoking, current		12	(36)	339	(32)	0.250
Statin use		14	(42)	376	(36)	0.592
Prevalence of SAH		12	(36)	427	(41)	0.749

ECAA = extracranial carotid artery aneurysm, n = number, sd = standard deviation, ADPKD = autosomal dominant polycystic kidney disease, IA = intracranial aneurysm, SAH = subarachnoid hemorrhage.

* indicates *p* < 0.05.

^a^ Combined with crude data from *Pourier et al*. [1]

**Table 3:** Multiple logistic regression analysis for ECAA prevalence.

Characteristics	Complete case analysis n = 1048_a_		
	**POR**	**[95% CI]**	***p***
Age	0.98	[0.95–1.01]	0.105
Male gender	**2.25**	**[1.09–4.64]**	0.028^*^
Origin of cohort	**2.41**	**[1.15–5.06]**	0.020^*^
Hypertension	1.41	[0.67–2.99]	0.368
Diabetes	0.58	[0.13–2.57]	0.471
Statin use	1.11	[0.51–2.39]	0.800

ECAA = extracranial carotid artery aneurysm, n = number, POR = prevalence odds ratio, CI = confidence interval.

* indicates p < 0.05

_a_ Combined with crude data from Pourier et al. [1]

The present study shows that approximately 1 out of 20 Finnish IA patients had an ECAA, which was more than twice the co-prevalence of the Dutch cohort. [1] After adjusting for confounding, origin of country and male gender remained as independent predictors for ECAA co-prevalence. Additionally, one out of four ECAAs in the Finnish cohort was located around the carotid bifurcation, and would have been missed if total carotid imaging was not performed (Fig 3).

Co-prevalence of aneurysms in different types of arteries has been widely reported in literature. Both co-existence of central abdominal aortic and thoracic aneurysms, [20,21] and peripheral aneurysms like iliac, femoral or popliteal are described. [10–13] Over the years, evidence for a common pathway for both IA and aortic and/or thoracic aneurysms has been raised. [14,15,22,23] The co-prevalence of IA and ECAA does fit in this scientific field, although our present research does not provide clear answers why these aneurysms share co-existence. Age, size of aneurysm, current smoking, [14] hypertension [15], and male gender [12] were proposed as clinical predictors for aneurysm co-prevalence. In the present study, only male gender and Finnish origin remained as clinical predictors for ECAA co-prevalence. Differences in cardiovascular risk profile between our studied cohorts (Table 1) seem not to explain why the Finnish origin is associated with ECAA co-prevalence (Table 2), though might influence partially. Types of scanners were comparable in both cohorts, thus differences in imaging technologies have been ruled out. Although the number of patients that underwent arterial catheterization by DSA prior to the ECAA diagnosis was comparable in patients with IA and ECAA (42% Dutch vs 61% Finnish, *p* = 0.261), iatrogenic dissections by the intraluminal intervention may have contributed to a higher rate of ECAAs in both cohorts. Most of iatrogenic pseudo-aneurysms have a benign course and tend to dissolve over time, therefore this influence is expected to be low. A genetic comparison in aneurysm related genes [22,23] may elucidate why the Finnish origin seems to be associated with higher ECAA co-prevalence, though obtaining a sufficient sample size would be challenging. In this way, the incidence of connective tissue disorders could be measured simultaneously.

The present study pointed out that if the carotid bifurcation is included within standard imaging protocol, almost every ECAA in an IA patient is detected (Fig 3). Hence, we propose to include imaging of the carotid bifurcation in IA patients as standard practice to identify ECAA in these patients (*e*.*g*. CTA stroke or carotids). In the Finnish cohort, 16% of patients faced ECAA-related symptoms like cerebral ischemia or nerve deficits (S1 Table). As IA-related symptoms tend to be more dominant and permanent, this number might be underestimated. Although it is still unclear which ECAA-related symptoms an individual patient might encounter, [2,4,6,7,8] monitoring of growth or configuration of the ECAA is necessary to detect alterations and prevent potential cerebral ischemia in terms of (sub)clinical infarcts and white matter lesions, and the potential loss of the ipsilateral blood supply by the carotid artery. The largest ECAA review to date [2] showed that 38% of treated ECAA patients were presented with stroke. This is of interest in patients who are already at high-risk of developing neurodegenerative diseases over-time. With a reasonable follow-up scheme, therapy decision making in IA and ECAA patients can be performed adequately.

Some limitations need to be addressed. First, although we used common definitions [17] for proximal and distal carotid artery as well as around the carotid bifurcation, one might find this definition arbitrary. As our research had to anticipate on different scan limits on CTA/MRA despite the complete carotid artery imaging protocol, a breakdown of the carotid artery was necessary. Moreover, the indication for complete carotid imaging might have led to residual confounding in which we are unable to correct for in this retrospective study. A more precise estimate of the co-prevalence of IA and ECAA is obtained by scanning all IA patients with similar scan modality, limits and study period. Nevertheless, the present study reflects clinical practice in two large tertiary referral centers in Europe and is therefore highly applicable to IA standard care. Also, comorbidities were defined in a pragmatic way, since databases were combined. Although all CT scanners were 64-slice, thin-slice images were not always available for review due to local hospital storage. Small aneurysms <3.0 millimeter could have been missed and not included in our present analysis, but this is the case for both the Finnish and Dutch cohort. Lastly, due to the rarity of ECAA, a large sample size is not feasible. Hence, we performed our multiple logistic regression analysis conventionally despite our event rate. Potential over- or under fitting of our model cannot be ruled out. Ideally, collaborations in the international ECAA registry (www.carotidaneurysmregistry.com) [8] are needed to increase sample sizes and firmly indicate potential predictors for ECAA presence.

## Conclusions

Co-prevalence of ECAA in IA patients was twice as high in another European cohort, with both origin of country and male gender as independent predictors. Approximately one out of four ECAAs would be missed if the carotid bifurcation was not imaged, hence we propose to always include imaging of the carotid bifurcation as the gold standard technique to identify ECAA in IA patients.

## Supporting information

S1 File Detailed methods Pourier et al. [1].
(PDF)

S2 File Supporting dataset. (XLSX)

S1 Table Aneurysm characteristics of identified 36 ECAAs in the Finnish cohort. Data are presented as n = number of aneurysms (%) unless otherwise indicated. ECAA = extracranial carotid artery aneurysm, TIA = transient ischemic attack, mm = millimeter, IA = intracranial aneurysm.
(PDF)
