# Supplementary material for: Co-prevalence of extracranial carotid aneurysms differs between European intracranial aneurysm cohorts
Source: PLoS One. 2020 Jan 23;15(1):e0228041. doi: 10.1371/journal.pone.0228041 (PMC6977743; doi:10.1371/journal.pone.0228041)
Supplement: S1 File — [1]. (PDF) [file pone.0228041.s001.pdf]

S2 Detailed methods Pourier et al. [1]

All consecutive patients with an intracranial aneurysm (IA) admitted to or seen at the outpatient clinic of the Department of Neurology and Neurosurgery of the University Medical Center Utrecht, the Netherlands, were eligible in the study of Pourier et al. [1]. Patients were included from 1978 up to 2015, with both ruptured and unruptured IAs. Patients were excluded in case of absence of an IA, if the intracranial hemorrhage was perimesencephalic or due to a trauma without a clear IA, or if the IA was associated with an arteriovenous malformation or dural fistula.

Two authors reviewed all available radiological imaging, divided in complete carotid imaging (from aortic arch up to skull base) and partially carotid imaging (one-sided or if only the distal carotid arteries were depicted from the second cervical vertebra i.e. CTA brain). All available carotid imaging was screened for the co-existence of an extracranial carotid artery aneurysm (ECAA). ECAA was defined as a fusiform dilation of the arterial diameter of ≥150% compared with the normal carotid artery diameter, for saccular aneurysms any size was accepted.

Reference: 1. Pourier VEC, van Laarhoven CJHCM, Vergouwen MDI, Rinkel GJE, de Borst GJ. Prevalence of extracranial carotid artery aneurysms in patients with an intracranial aneurysm. PLoS One. 2017;12: e0187479. doi:10.1371/journal.pone.0187479
