# Supplementary material for: Co-prevalence of extracranial carotid aneurysms differs between European intracranial aneurysm cohorts
Source: PLoS One. 2020 Jan 23;15(1):e0228041. doi: 10.1371/journal.pone.0228041 (PMC6977743; doi:10.1371/journal.pone.0228041)
Supplement: S1 Table — Data are presented as n = number of aneurysms (%) unless otherwise indicated. ECAA = extracranial carotid artery aneurysm, TIA = transient ischemic attack, mm = millimeter, IA = intracranial aneurysm. (PDF) [file pone.0228041.s003.pdf]

**S1 Table.** Aneurysm characteristics of identified 36 ECAAs (both complete and incomplete carotid imaging) in the Finnish cohort.					
		**Fusiform n = 26**		**Saccular n = 10**	
		**n**	**(%)**	**n**	**(%)**
**ECAA related symptoms**					
	Ipsilateral ischemic stroke / TIA	1	(4)	1	(10)
	Horner's syndrome	1	(4)	1	(10)
	Cranial nerve deficit	2	(8)	1	(10)
	Asymptomatic	13	(50)	4	(40)
	Unknown	*9*	*(35)*	*3*	*(30)*
**Presumed etiology in radiology report**					
	Dissection	6	(23)	2	(20)
	Connective tissue disease	1	(4)	-	
	Atherosclerosis	4	(15)	-	
	Iatrogenic	1	(4)	1	(10)
	Unknown	*14*	*(54)*	*7*	*(70)*
**Side**					
	Left	18	(69)	5	(50)
	Right	8	(31)	5	(50)
**Size in mm, median (range)**		9.4	(7.4 – 35.0)	4.3	(2.0 – 7.2)
**Location IA cerebral circulation**					
	Anterior circulation	22	(85)	8	(80)
	Posterior circulation	3	(12)	2	(20)
	Both anterior & posterior circulation	1	(4)	-	
**Multiple IA**		5	(19)	1	(10)
**Fusiform IA**		1	(4)	1	(10)
Data are presented as n=number of aneurysms (%) unless otherwise indicated. ECAA = extracranial carotid artery aneurysm, TIA = transient ischemic attack, mm = millimeter, IA = intracranial aneurysm.					
